# Supplementary figures and images for: Topography of hippocampal connectivity with sensorimotor cortex revealed by optimizing smoothing kernel and voxel size
Source: PLoS One. 2021 Dec 7;16(12):e0260245. doi: 10.1371/journal.pone.0260245 (PMC8651104; doi:10.1371/journal.pone.0260245)

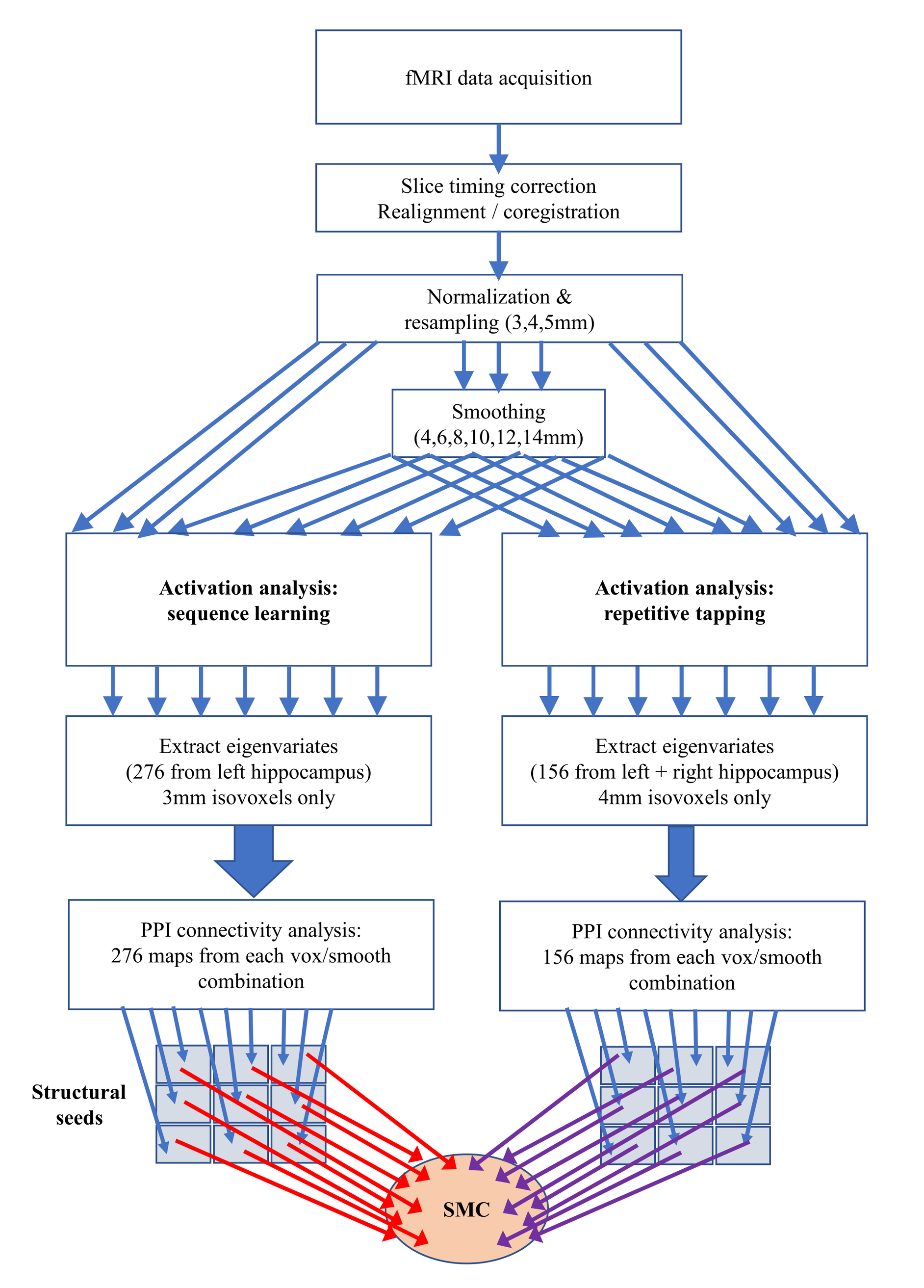

Supplement: S1 Fig — Data was resampled with 3mm, 4mm, and 5mm isovoxels following normalization. For each voxel size, data was smoothed and analyzed separately across a range of smoothing kernels. (TIF) [file pone.0260245.s001.tif]

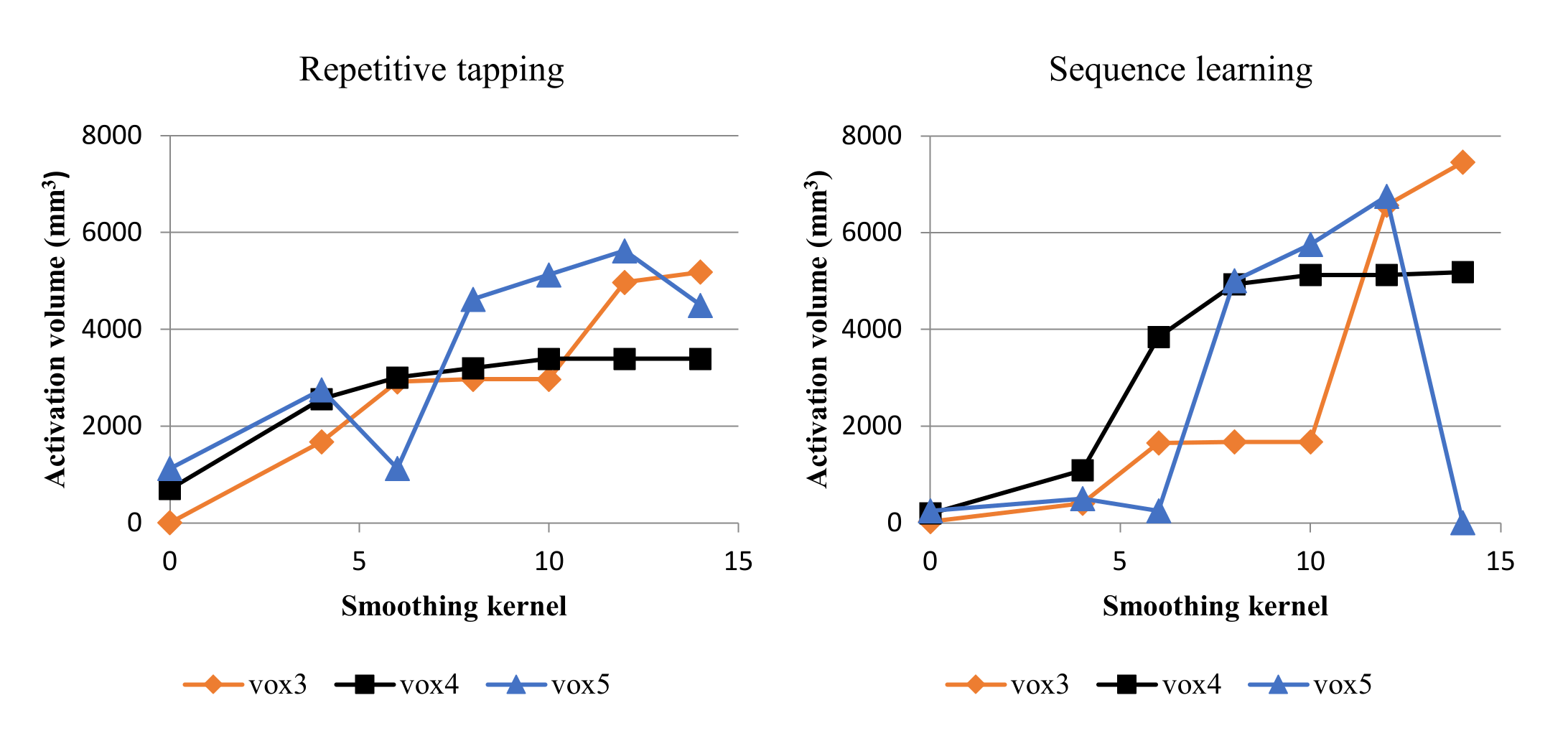

Supplement: S2 Fig — Within limits, larger smoothing kernels generated larger volumes of activation during group analysis regardless of voxel size. Maximal volume was typically generated with smoothing kernels of 8-12mm. (TIF) [file pone.0260245.s002.tif]

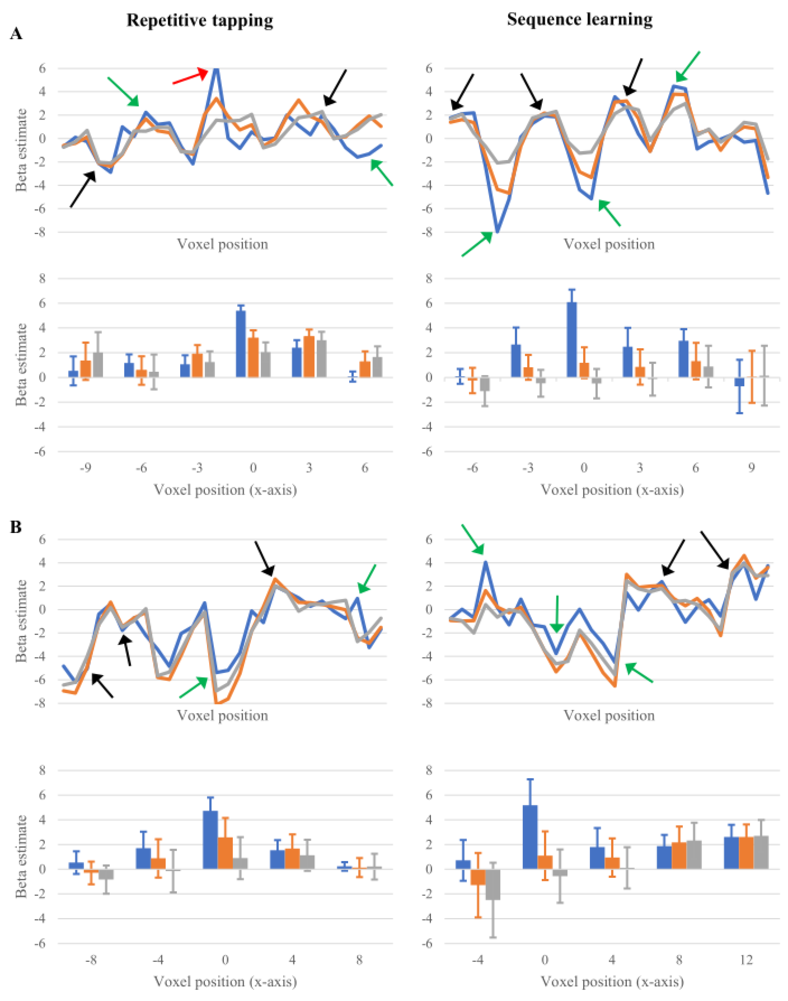

Supplement: S3 Fig — (A) Effects of smoothing on activation magnitude from adjacent 3mm isovoxels; no smoothing (blue) and smoothing kernels of 6mm (orange) and 10mm (gray) are shown. Depending on similarity of responses in neighboring voxels, smoothing had minimal effects on peak activation or deactivation (black arrows) or greatly affect response amplitude (green arrows); the red arrow shows peak activation for the entire hippocampus. Summarized across all individuals, activation was elevated 9-12mm surrounding the peak, maintained with smoothing during repetitive tapping but eliminated during sequence learning. (B) Effects of smoothing on activation magnitude from adjacent 4mm isovoxels. Effects of smoothing again depended on similarity of responses in neighboring voxels; before smoothing, activation was elevated 12-16mm surrounding the peak, reduced then eliminated with larger smoothing kernels for both repetitive tapping and sequence learning. (TIF) [file pone.0260245.s003.tif]
