# Supplementary material for: Topography of hippocampal connectivity with sensorimotor cortex revealed by optimizing smoothing kernel and voxel size
Source: PLoS One. 2021 Dec 7;16(12):e0260245. doi: 10.1371/journal.pone.0260245 (PMC8651104; doi:10.1371/journal.pone.0260245)
Supplement: Attachment — Submitted filename: Response to Reviewers.docx [file pone.0260245.s005.docx]

PONE-D-20-38639
Topography of hippocampal connectivity with sensorimotor cortex revealed by optimizing smoothing kernel and voxel size
PLOS ONE

Responses to reviewer comments are provided below in italics.

Reviewer #1: I thank the author for the thorough response to my previous comments. I appreciated the clarification of several of the comments and I would like to comment on several points that this discussion has brought up to light.

The addition of a validity and limitation section is helping to frame the use of the result from this investigation. However, I still believe such an article is difficult to apply to other datasets of potential reader as it mainly relies on specific analysis outputs, and do not provide a measure of reproducibility. This could influence reader to test all range of parameters to obtain the largest activation without questioning false negative.

*There are limits to what can be done to generalize results from one study, but in the absence of a well-defined region of activation to justify seed placement, there are advantages to using the approach described in this study. I have added to the introduction a statement that succinctly summarizes the purpose of this paper: “**The purpose of the methodological component of this study is not to identify optimal parameters for every case, but to demonstrate that parameters commonly used in hippocampal studies may not be optimal for connectivity studies with cortex.”*

*Informally, the methods and parameters identified can be applied to other studies. Using the optimal parameters described here, I have run PPI analyses on tasks from previous studies, finding found noteworthy results (e.g., connectivity with task-related brain areas during tasks such as Stroop, language tasks emphasizing different linguistic components, several movement tasks, and memory tasks). Data acquisition from these studies ranged from 3mm to 4mm isovoxels; after finding no discernible differences in summary figures, I decided to use 4mm isovoxels because of major differences in processing demands and disk space requirements. (Using both voxel sizes, the current study exceeded my 1TB disk space, requiring three computers to complete.) I’ve been hesitant to submit findings from these other studies until the current paper is published, due to strong opposition I’ve faced from previous reviewers over my choice of processing parameters.*

*Neuroimaging studies are not designed to exclude false negatives. A negative finding does not mean that an effect is not there, only that observed differences could statistically occur by chance (i.e., greater than a 5% probability). This explains why statistical standards used in research reports are not applied in the clinical setting (e.g., presurgical planning for brain tumor patients), where consequences of a false-negative finding are far more devastating than a false-positive. Researchers utilize those parameters likely to produce positive results; this study provides a method for evaluating connectivity throughout the hippocampus when activation is not available to guide seed placement.*

Seeing that 3mm and no smoothing is more advantageous in an individual (for activation) versus lower resolution and higher smoothing for group analysis makes me wonder of the reproducibility of the results and the trend behind this optimization choice. Could a summary of the same individual analysis be provided for the dataset, in order to visualize variability in the sample and not referring only to the most representative subject? Single subject analyses are also relevant for lab focusing on specific subject, precision medicine and case studies. Additionally, or alternatively, variance map in activation result across the individuals result could help understand the change in effect between resolution and smoothing.

*A summary of individual activation analysis has been added in a supplementary figure (S3 Fig). The mean magnitude of activation and standard error from all individual was plotted at the activation maximum and adjacent voxels, both for 3mm and 4mm isovoxels across both motor tasks. Before smoothing, the area of activation was comparable for both (12-16mm diameter). This activation disappeared with smoothing for 4mm isovoxels in both tasks and for 3mm isovoxels in the sequence learning task; thus, the advantage for finding activation with 3mm isovoxels depended on the analysis. Plotting beta estimates for activation across voxels at three smoothing kernels (0, 6, and 10) showed changes in activation amplitude with smoothing where adjacent voxels show sharp gradients in activation, but not where adjacent voxels responded the same. Consistent with the 12mm activation radius surrounding the maxima, this suggests a 10mm smoothing kernel does not exceed the width of a functional unit of activity in the hippocampus; instead, effects are reduced at transitional areas where the activity of nearby voxels differ sharply.*

*Emphasis on activation analysis on an individual level in this study seems misplaced, however, given that A) hippocampal activation was not observed during group analysis, B) activation maxima were shown not to generate connectivity consistent with functional seeds, and C) the topography shown for connectivity would not be evident from activation maxima. Connectivity represents moment-to-moment changes in activity that were not captured by activation analysis.*

Can a summary be provided for half split reproducibility of group analysis, in order to strengthen the choice of resolution X smoothing effect and show a framework for other reader that would need to do this investigation to select their own optimal parameter for their specific task designs?

*A half split reproducibility of group analysis works best for large samples, unlike the sample size from this study.*

As an additional minor comment, the first paragraph of the validity and limitation is lacking a recall of reference.

*A reference has been added.*

Reviewer #2: I am happy with all the responses made by the author apart from one major comment related to the resampling of voxels sizes. The author stated that "As noted above, the voxel size at acquisition is comparable to other studies; resampling is not done until the normalisation step, and is thus unaffected by prior preprocessing steps (such as slice timing correction and realignment). Acquiring separate sets of data with high resolution and low-resolution voxels would introduce additional sources of variability that would confound the findings."

I disagree with this response since resampling or "playing" with the data set is :
- not a common practice in the field and not everyone would test it
- done on an already low resolution dataset

*Labs do not commonly resample at multiple resolutions, as they are not trying to optimize parameters -- and based on their past experience and results, they assume their parameters are fine. As detailed in my previous cover letter, most studies actually do resample at a higher resolution than what was acquired, as greater resolution during acquisition would limit brain coverage or increase TR. The current study does not undercut results from previous studies, but simply points out that findings may be overlooked with suboptimal processing parameters.*

Therefore, the comment made by the author regarding acquiring other dataset with high or low res may add sources of variability among subjects could be true. Nonetheless, this could be overcome easily with taking into considerations these changes into a perfectly balanced statistical model and in theory would result in a better estimation than the current methods and findings of this paper.

I would not reject the paper because of this point. However, the author should clearly state this in the limitation part. Until then, I am happy to recommend accepting this paper.

*The following statement has been added: “Hippocampal connectivity studies acquired with different resolutions and involving other cortical areas are needed to elucidate how widespread the optimal parameters from this study may be applied.”*

I also suggest the author to re-consider the conclusion of this paper where it stated "This study shows that processing fMRI data with a larger voxel size (4mm) and smoothing kernel (8-10mm) can improve sensitivity..." This recommendation is I think overestimated given the small size of the hippocampus and the type of the acquired manipulated dataset. I hope the conclusion to be more realistic and precisely give a statement with regard to the used manipulated dataset and applied methodology. Also what was the sensitivity factor (golden reference/standard) that based on which the author reached to this conclusion.

*The conclusion has been modified as shown below in boldface, making it clear that improved sensitivity from these processing parameters occurred here but may not occur in all studies. There is no “gold standard” in the literature for measuring sensitivity, but demonstrating topography in hippocampal connectivity with SMC required these larger smoothing kernels and voxel sizes, as smaller parameters failed to demonstrate connectivity across the breadth of the hippocampus. As such, these parameters were more sensitive in this study, since sensitivity in fMRI analyses is defined as “the probability of detecting [a property] given it exists” (Hopfinger et al, 2000: A Study of Analysis Parameters That Influence the Sensitivity of Event-Related fMRI Analyses).*

*Conclusion: “This study shows that processing fMRI data with a larger voxel size (4mm) and smoothing kernel (8-10mm) can,* ***at least in some cases****, improve sensitivity to hippocampal activity and its connectivity with cortical areas; optimizing these processing parameters* ***in this study*** *uncovered topography in hippocampal connectivity along two axes.”*
