# Supplementary material for: Topography of hippocampal connectivity with sensorimotor cortex revealed by optimizing smoothing kernel and voxel size
Source: PLoS One. 2021 Dec 7;16(12):e0260245. doi: 10.1371/journal.pone.0260245 (PMC8651104; doi:10.1371/journal.pone.0260245)
Supplement: Attachment — Submitted filename: Response to Reviewers.docx [file pone.0260245.s006.docx]

PONE-D-20-38639
Topography of hippocampal connectivity with sensorimotor cortex revealed by optimizing smoothing kernel and voxel size
PLOS ONE

Responses to reviewer comments are provided below in italics.

Reviewer #1: I thank the author for the changes in introduction and conclusion and the addition of the supplementary figure. It gives a better frame for the reader on the aim of the article. While the sample size brings limitation to the generalization of this optimization, this article provides a rethinking frame for detecting appropriate area for connectivity analysis depending on data resolution and context. To the extent of what the data use in this investigation can offer, the case of activation-connectivity relationship is illustrated.

This article is a methodological case example. However any study using this optimization method cannot do so to the expense of a meaningful description of the variability and effect of confounds on their resulting connectivity maps and inferences. If this is clear, I have no further comment.

*This article may be considered a methodological case example, demonstrating that processing parameters commonly used to study activation within the hippocampus may not be optimal for identifying its influence on cortical areas. Regardless of methodology, however, a meaningful description of the variability and effect of confounds on the resulting connectivity maps and inferences should always be considered; connectivity measurements reflect the net influence of all factors* *affecting hippocampal activity and its connections.*

Reviewer #2: [No comments]
